# Filtrate of *Phellinus linteus* Broth Culture Reduces Infarct Size Significantly in a Rat Model of Permanent Focal Cerebral Ischemia

**DOI:** 10.1093/ecam/nen091

**Published:** 2011-04-06

**Authors:** Sakiko Suzuki, Takakazu Kawamata, Yoshikazu Okada, Tomonori Kobayashi, Tomoyuki Nakamura, Tomokatsu Hori

**Affiliations:** ^1^Department of Neurosurgery, Tokyo Women's Medical University, 8-1 Kawadacho, Shinjuku-ku, Tokyo 162-8666, Japan; ^2^Applied Fungi Institute, IBI Corporation, Yamanashi, Japan

## Abstract

*Phellinus linteus*, a natural growing mushroom, has been known to exhibit anti-tumor, anti-inflammatory, anti-allergic and anti-oxidant effects. Aiming to exploit the neuroprotective effects of *P. linteus*, we evaluated its effects on infarct volume reduction in a rat model of focal cerebral ischemia. Male Sprague-Dawley rats were subjected to right middle cerebral artery occlusion. Filtrate of *P. linteus* broth culture (various doses), fractionated filtrate (based on molecular weight) or control medium was administered intraperitoneally to rats before or after ischemia induction. Rats were killed at 24 h after the stroke surgery. Cortical and caudoputaminal infarct volumes were determined separately using an image analysis program following staining with 2,3,5-triphenyltetrazolium chloride. Significant cortical infarct volume reductions were found in the pre-treatment groups (30 and 60 minutes before onset of cerebral ischemia) compared with the control group, showing dose dependence. Posttreatment (30 minutes after ischemic onset) also significantly reduced cortical infarct volume. Furthermore, the higher molecular weight (≥12 000) fraction of the culture filtrate was more effective compared with the lower molecular weight fraction. The present findings suggest that *P. linteus* may be a new promising approach for the treatment of focal cerebral ischemia, with the additional benefit of a wide therapeutic time window since significant infarct volume reduction is obtained by administration even after the ischemic event. Our finding that the higher molecular weight fraction of the *P. linteus* culture filtrate demonstrated more prominent effect may provide a clue to identify the neuroprotective substances and mechanisms.

## 1. Introduction

*Phellinus linteus* (Berk. Et Curt.) Teng has long been used as traditional oriental medicine, and is known as “song gen" in Chinese and “meshimakobu" in Japanese. Investigations of the pharmacologic effects of *P. linteus* were started in Japan about 40 years ago, and this mushroom showed the strongest anti-tumor effects compared to other mushrooms [1]. *P. linteus* is now attracting attention for its anti-inflammatory, anti-allergic, anti-tumor and anti-oxidant effects [2–4].

Tea catechins are known to act as antioxidants by scavenging free radicals and chelating metal ions [5, 6]. Like tea catechins, caffeic acid also possesses a catechol moiety in its structure. Caffeic acid may exhibit antioxidant effect by the same mechanism as that of tea catechins. Caffeic acid was extracted from the culture of *P. linteus* mycelia [7]. It would appear that the antioxidant effect of *P. linteus* may be due to caffeic acid.

Cerebral infarction is caused by death of brain tissues mainly due to thromboembolic occlusion of a cerebral artery. The patient suffered from sequelae including hemiparesis, sensory disturbance, aphasia, memory disturbance and visual disturbance. The severity of the sequelae depends on not only the area of the ischemic focus but also the area of penumbra. Some reports indicated that free radicals generated during ischemia play a prominent role in neural damage [8, 9]. In this context, studies have shown that eliminating active oxygen has strong cerebroprotective effect on brain ischemia [10].

The effects of *P. linteus* on central and peripheral nervous systems have not been reported. In the present study, we pre- or post-treated middle cerebral artery occlusion rat models with the filtrate of *P. linteus* broth culture to estimate its cerebroprotective effect. The study was designed to elucidate the effective dose, the therapeutic time window and the effective fraction of the filtrate.

## 2. Materials and Methods

### 2.1. Surgical Procedures of Middle Cerebral Artery (MCA) Occlusion Models

The experimental protocol used in this study was approved by the Ethics Committees for Animal Experimentation at Tokyo Women's Medical University. Male Sprague-Dawley rats (8-week old and weighing 270–330 g) were maintained on a regulated 12-h light:dark cycle and allowed free access to water and food. Rats were anesthetized with 1 mL kg^−1^ phenobarbital sodium by intraperitoneal injection. Body temperature was maintained at 37 ± 0.5°C using a feedback-regulated heating pad during the procedure. The right femoral artery was cannulated for continuous monitoring of arterial blood pressure and arterial blood gas levels (PaO_2_, PaCO_2_, pH and base excess), hematocrit and glucose level. Arterial blood gas and glucose levels were analyzed before the ischemic surgery and immediately after the ischemia.

A modified, permanent MCA occlusion model by clipping the proximal right MCA was adopted in this study [11]. In brief, a 10-mm linear skin incision and a muscle incision were made between the lateral corner of the right eye and the external auditoty orifice. The temporal bone between the orbital fissure and the foramen ovale, especially the temporal base, was drilled and removed. The dura was dissected and opened to approach the right MCA. The zygomatic arch, orbital contents and the facial nerve were preserved during the whole procedures as reported previously by our group [11–15]. A pial dissection along the MCA was performed to provide space for the clip. The proximal side of MCA (just proximal to the olfactory nerve) was occluded by applying the clip [11]. The MCA was electrocoagulated from the point of clipping to just proximal to the level of the inferior cerebral vein with a bipolar diathermy and transected using a surgical microscope [11–15].

### 2.2. Experimental Protocols

*Phellinus linteus* strain PL-08 was used in the present study. The strain is maintained at IBI Co., Ltd, Yamanashi, Japan. The mycelia of strain PL-08 were cultured for 45 days in a broth medium containing 4% glucose, 0.3% yeast extract, 0.3% polypeptone, 0.05% potassium dihydrogen phosphate and 0.05% disodium hydrogen phosphate in distilled water, adjusted to pH 5.5. For culture, a 5-L fermenter was used and incubated at 25°C in a dark chamber. Sterilized air was passed into the fermenter through a filter with pore size 0.22 *μ*m (Millez-FG50; Millipore, USA) at a flow rate of 1 L min^−1^. The 45-day broth culture was filtered and divided into mycelia and culture filtrate. The culture filtrate was used as the test substance in the present study.

Four percent and 20% culture filtrates were prepared by diluting the neat filtrate in distilled water. Appropriate volumes of the two culture filtrates were administered to obtain various doses. As a preliminary screening for the molecules responsible for the neuroprotective effect, we fractionated the 20% culture filtrate into two fractions at a cutoff molecular weight (MW) of 12 000. For the same purpose, we also prepared a glycoprotein extract from the 20% culture filtrate. Therefore, a total of three fractions of *P. linteus* culture filtrate were tested in the present study and designated Groups 7 (MW < 12 000), 8 (MW ≥ 12 000) and 9 (glycoprotein). The *P. linteus* culture filtrate was prepared (20%) and three volumes of ethanol were added. The resulting suspension was lyophilized. For administration, the powder was dissolved in phosphate buffer (20 mg mL^−1^).

The rats were assigned randomly to 12 experimental groups (nine PL-08 treatment groups and three control groups). Before or after experimental induction of focal cerebral ischemia, the culture filtrate (treatment groups) or medium (control groups) was administered intraperitoneally to the rats according to the protocol as follows: Group 1: 1.5 mL of 20% culture filtrate at 60 min before ischemic onset (*n* = 5), Group 2: 2.5 mL of 20% culture filtrate at 60 min before ischemic onset (*n* = 5), Group 3: 2.5 mL of 4% culture filtrate at 30 min before ischemic onset (*n* = 5), Group 4: 1.5 mL of 20% culture filtrate at 30 min before ischemic onset (*n* = 7), Group 5: 2.5 mL of 20% culture filtrate at 30 min before ischemic onset (*n* = 7), Group 6: 1.5 mL of 20% culture filtrate at 30 min after ischemic onset (*n* = 7), Group 7: 2.5 mL of fraction with MW < 12 000 at 30 min before ischemic onset (*n* = 6), Group 8: 2.5 mL of fraction with MW ≥ 12 000 at 30 min before ischemic onset (*n* = 6), Group 9: 2.5 mL of glycoprotein extract at 30 min before ischemic onset (*n* = 8), Control 1: control for Group 3; 2.5 mL of 4% medium at 30 min before ischemic onset (*n* = 5), Control 2: control for Groups 1, 4 and 6; 1.5 mL of 20% medium at 60 min before (for Group 1), 30 min before (for Group 4) or 30 min after (for Group 6) ischemic onset (*n* = 11), and Control 3: control for Groups 2, 5, 7, 8 and 9; 2.5 mL of 20% medium at 60 min before (for Group 2) or 30 min before (for Groups 5, 7, 8 and 9) ischemic onset (*n* = 15). We considered the three control groups necessary because the differences in glucose and other compositions in the inocula may affect the infarct volume.

Twenty four hours after the stroke surgery, the animals were killed with an overdose of intraperitoneal phenobarbital sodium and decapitated.

### 2.3. Evaluation of Infarct Volume

Brain samples were obtained 24 h after the surgery. Seven coronal sections 2-mm thickness were cut and immediately stained with 2% 2,3,5-triphenyltetrazolium chloride (TTC) [16, 17]. The cerebral infarction area in the brain slice was measured with an image analysis program (Beta 4.0.2 of Scion Image). To compensate for the effect of brain edema, the infarct volume was determined using an indirect method: infarct area =  (area of the intact contralateral hemisphere) − (area of the intact ipsilateral hemisphere) [18]. The infarct volume was expressed as a percentage of the intact contralateral hemispheric volume. Infarct volume of the cortex and caudate putamen was measured separately. Infarct volume in the cortex was measured using slices 2 through 5 and infarct volume in the caudate putamen was measured using slices 3 and 4.

### 2.4. Statistical Analysis

The researchers who performed intraperitoneal injection, ischemic surgery and infarct volume measurement were blinded to which groups the animals were assigned until all data had been collected. Values presented, in this study, are expressed as mean ± SD. One-way analysis of variance (ANOVA) followed by *post hoc* Tukey-Kramer test was used to determine the statistical significance of differences in physiological variables and infarct volumes among all the groups. A *P*-value <.05 was considered statistically significant.

## 3. Results

### 3.1. Physiological Parameters

There were no significant differences among all groups in body temperature; arterial blood pressure; arterial blood gas levels including PaO_2_, PaCO_2_, pH and base excess; hematocrit; and blood glucose.

### 3.2. Infarction Volume

#### 3.2.1. Overall Analysis

**Figure 1:**
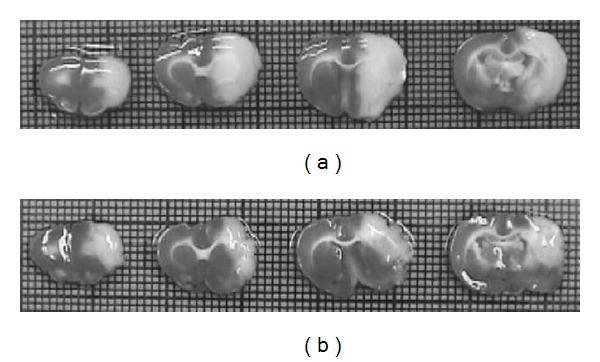
Representative 2,3,5-triphenyltetrazolium chloride-stained brain sections following right proximal MCA occlusion in the control group ((a); Control 3) and the *Phellinus linteus* culture filtrate-treated group ((b); Group 5). Infarct volumes in the *P. linteus*-treated group are smaller than those in control group. Coronal sections are obtained from locations +4.5, +2.5, +0.5 and −1.5 mm relative to the bregma in each group.

As described previously [12–16] infarcts produced by occlusion of the proximal MCA involve the dorsolateral cerebral cortex and underlying subcortical structures in the ipsilateral hemisphere (Figure 1). Specifically, infarcts involve regions of the cortex controlling sensorimotor functions of the contralateral limbs, as well as the caudate putamen (Figure 1).

In the analysis of Group 3 (2.5 mL of 4% culture filtrate at 30 min before ischemic onset) compared with Control 1, no differences in infarct volume in the cortex (60.6 ± 9.2% versus 60.1 ± 4.4%) and the caudate putamen (40.7 ± 10.6% versus 42.5 ± 13.8%) were observed.

**Figure 2:**
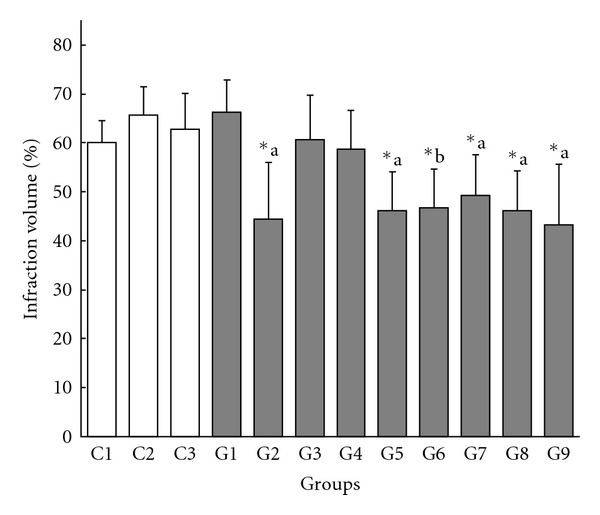
Effect of administration of *Phellinus linteus* culture filtrate on infarct volume in cortex. Infarct volumes (%) are presented as mean ± SD, and are calculated by the “indirect method". C1–3 are control groups and G1–9 are *P. linteus*-treated groups. See Methods for explanations of the 12 groups. *a: Statistically significant differences, C3 versus G2, G5, G7, G8 and G9. *b: Statistically significant differences, C2 versus G6.

**Figure 3:**
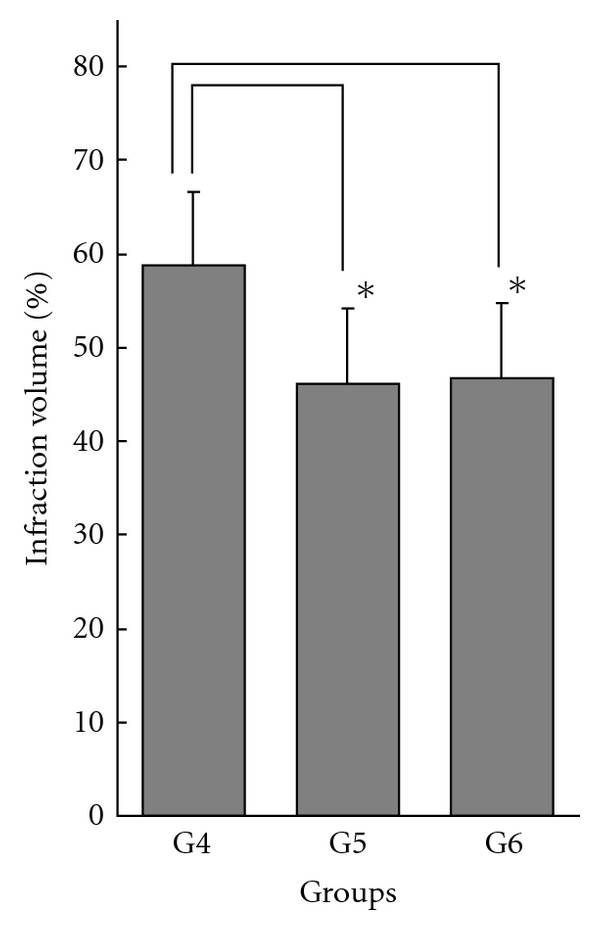
Effect of administration of *Phellinus linteus* culture filtrate on infarct volume in cortex: comparing pre-ischemic treatment (30 min before ischemic onset) and post-ischemic treatment (30 min after ischemic onset). Infarct volumes (%) are presented as mean ± SD, and are calculated by the “indirect method". G4 (low dose) and G5 (high dose) are pre-treatment groups and G6 (low dose) is post-treatment group. Cortical infarct volume is significantly reduced in the group administered the filtrate 30 min after ischemic onset (G6) compared with the group administered the same dose 30 min before ischemic onset (G4). *Statistically significant differences.

In the analysis of Groups 1, 4 and 6 compared with Control 2, significant difference in cortical infarct volume was detected (*F* = 12.04, *P* < .0001; 66.2 ± 6.6%, 58.7 ± 7.9%, 46.7 ± 8.0% and 65.7 ± 5.8%, in Groups 1, 4, 6 and Control 2, resp.) (Figure 2). Furthermore, significant differences in cortical infarct volume were observed when comparing Group 6 (1.5 mL of 20% culture filtrate at 30 min after ischemic onset) with Control 2 and Group 4 (the same dose at 30 min before ischemic onset) (one-way ANOVA followed by *post hoc* Tukey-Kramer test) (Figures 2 and 3). These results indicated that post-ischemic treatment with culture filtrate was effective in reducing infarct volume compared with control, and the effect was also observed when compared with pre-ischemic treatment. There were no significant differences in infarct volume in the caudate putamen (49.0 ± 17.5%, 42.3 ± 12.0%, 44.6 ± 15.2% and 48.5 ± 11.8% in Groups 1, 4, 6 and Control 2, resp.).

In the analysis of Groups 2, 5, 7, 8 and 9 compared with Control 3, significant difference in cortical infarct volume was detected (*F* = 6.75, *P* = .0001; 44.4 ± 11.6%, 46.2 ±7.9%, 49.3 ± 8.3%, 46.2 ± 8.0%, 43.3 ± 12.3% and 62.8 ± 7.3%, in Groups 2, 5, 7, 8, 9 and Control 3, resp.) (Figure 2). Furthermore, significant differences in cortical infarct volume were observed when comparing the all five groups versus Control 3 (one-way ANOVA followed by *post hoc* Tukey-Kramer test) (Figure 2). There were no significant differences in infarct volume in the caudate putamen (40.2 ± 15.5%, 42.5 ± 12.6%, 38.5 ± 13.4%, 36.9 ± 14.8%, 36.1 ± 9.1% and 49.6 ± 9.1% in Groups 2, 5, 7, 8, 9 and Control 3, resp.).

Because there were no significant differences in cortical infarct size among the three control groups (Control 1, 2 and 3), the treatment groups could be compared directly as described below.

#### 3.2.2. Pre-Ischemic Treatment

**Figure 4:**
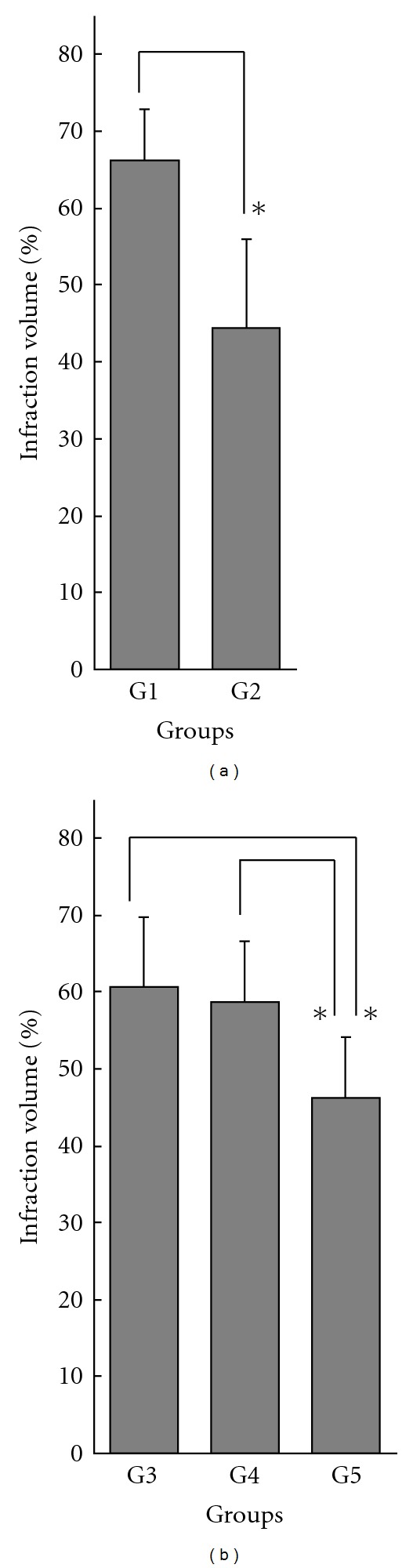
Effect of administration of *Phellinus linteus* culture filtrate on infarct volume in cortex: dose dependence in pre-ischemic treatment at 60 min (a) and at 30 min (b) before ischemic onset. Infarct volumes (%) are presented as mean ± SD, and are calculated by the “indirect method". G1: low dose, G2: high dose, G3: low dose, G4: medium dose, G5: high dose. For each treatment schedule, the highest dose (G2 and G5) shows significantly greater reduction of infarct volume. *Statistically significant differences.

According to the statistical analyses mentioned above, pre-ischemic treatment with the culture filtrate at 60 min (Groups 1 and 2) or 30 min (Groups 3, 4 and 5) before cerebral ischemia reduced cortical infarction significantly at the highest doses (Group 2 and Group 5) (Figures 2 and 4). There were no significant differences between treatments at 30 min and at 60 min before ischemia using the same doses (Group 1 versus Group 4 and Group 2 versus Group 5).

#### 3.2.3. Post-Ischemic Treatment

In Group 6, culture filtrate was administrated at 30 min after ischemic onset. This post-ischemic treatment was significantly effective in reducing the cortical infarct volume compared with control, and the effect was also significantly great when compared with pre-ischemic treatment using the same dose (Group 4), while there was no significant difference between Group 4 and control (Figures 2 and 3). Post-ischemic treatment at 30 min after ischemic onset using a lower dose showed similar effect as pre-ischemic treatment using a higher dose (Group 5 versus Group 6) (Figure 3).

#### 3.2.4. Dose Dependence

To examine dose dependence, we compared the cortical infarct volumes at each pre-treatment time. In the 60-min pre-treatment groups, there was a significant difference between Group 1 (lower dose) and Group 2 (higher dose) (*F* = 11.043, *P* = .0127) (Figure 4(a)). In the 30-min pre-treatment groups, there was a significant difference among the three groups in cortical infarct volume (Groups 3, 4 and 5; *F* = 5.858, *P* = .0123) (Figure 4(b)). The differences were significant when comparing Group 5 (highest dose) with Group 3 (lowest dose) and with Group 4 (medium dose) (one-way ANOVA followed by *post hoc* Tukey-Kramer test) (Figure 4(b)).

These results showed dose dependence in treatment with of *P. linteus* culture filtrate for cortical cerebral infarction.

#### 3.2.5. Fractionation of Culture Filtrate Based on Molecular Weight

When we fractionated the 20% culture filtrate into two fractions according to a MW cutoff of 12 000, 92.4% of the components were in the fraction with MW < 12 000 while only 7.6% were in the fraction with MW ≥ 12 000. Both fractions were similarly effective in reducing cortical infarct volume (49.3 ± 8.3% in Group 7 (MW < 12 000) and 46.2 ± 8.0% in Group 8 (MW ≥ 12 000)) (Figure 2) and there was no statistical difference between the two groups. Since the higher MW fraction gave significant effect despite containing a small portion of the components (7.6%), this result suggests a possibility that the higher MW fraction may contain a high proportion of active ingredients responsible for the infarct volume reduction effect.

#### 3.2.6. Glycoprotein Extract

The glycoprotein extract of the culture filtrate reduced cortical infract volume significantly (Group 9 in Figure 2). This result suggests that glycoprotein is one of the efficacious substances in the *P. linteus* culture filtrate which reduces cortical infarction.

## 4. Discussion

### 4.1. Reduction of Infarct Volume Using *P. linteus*

Focal ischemia was induced by clipping and coagulation of the MCA (modified Tamura model) in the present study. The results demonstrated that the 20% culture filtrate was effective to reduce the volume of brain ischemia compared with control. The ischemic volume reduction was greater in the cortex which is a region dominated by some collateral flow, than in the basal ganglia which is dominated by end arteries. The area of reduced ischemia in the cortex appeared to be identical to the ischemic penumbra that can be rescued with residual cerebral blood flow (collateral flow).

The present results demonstrated that treatment with the 20% culture filtrate 30 and 60 min before MCA occlusion effectively reduced the volume of brain ischemia. The neuroprotective effect in brain ischemia was dose-dependent. Furthermore, treatment with the culture filtrate 30 min after cerebral ischemic onset also gave prominent reduction of cortical infarct volume. From the clinical points of view, these results suggest a possibility that *P. linteus* culture filtrate is very promising in providing wide therapeutic time windows for the treatment of cerebral ischemia.

Although the present animal experiments showed that *P. linteus*, which is a natural spontaneous mushroom, significantly reduced cerebral ischemia in rats, the exact substances exhibiting neuroprotective effects have not been identified. As a preliminary screening for the pharmacological active substances in the culture filtrate, we fractionated the filtrate based on molecular weight. According to the present results, the fraction with higher MW may contain a greater proportion of active ingredients responsible for the reduction of cortical infarct volume. Moreover, the present results also suggest that glycoprotein had a significant effect in the reduction of cerebral infarct volume.

### 4.2. Mechanisms of Neuroprotective Effects of *P. linteus*

Based on the known medicinal action of *P. linteus*, antioxidant effect may be involved in the mechanism of neuroprotective effect in the ischemic brain. In a study that estimated the superoxide radical erasing effect of a culture of *P. linteus* by measuring superoxide radicals trapped with 5,5-dymethyl-1-pyrroline N-oxide (DMPO) by ESR spectra, a high superoxide radical erasing effect, that is to say, antioxidant effect, was recognized in the extract of *P. linteus* [7]. Additionally, fractionation studies were attempted to search for the constituents in fractions that exhibited intense superoxide radical scavenging activity. Caffeic acid was purified from the component with intense superoxide radical scavenging activity, which was twice as strong as that of vitamin C, known to be a potent antioxidant [7]. Moreover, the component that did not contain caffeic acid exhibited stronger antioxidant activity. Although there seemed to be other active ingredients with stronger antioxidant action than caffeic acid, it was impossible to isolate the other active ingredients because they were unstable and possibly copolymerized during the procedure of isolation.

Caffeic acid is the only active ingredient with antioxidant effect which was purified from the culture of *P. linteus* [7]. Also in a study that compared the hydroxyl radical scavenging effect of the mycelial extracts of twelve fungi, the extract of *P. linteus* had the strongest scavenging effect [7].

The catechol moiety, which is found in both caffeic acid and tea catechins, has the ability to provide electron to active oxygen and scavenger active oxygen. Previous studies have shown that compounds containing the catechol moitey exhibit significant hydroxyl radical scavenging effect, while other compounds have no such effect [19, 20]. In *P. linteus* culture, the antioxidant effect is attributed not only to caffeic acid that contains catechol moiety, but also to other unstable active ingredients yet to be isolated and identified.

Caffeic acid with MW of 180 is contained in the lower MW fraction when the culture filtrate of *P. linteus* is divided into two fractions at a MW cutoff of 12 000. In the present study, the higher MW fraction showed more effective reduction of ischemic area than the lower MW fraction. This result suggests that other ingredients having stronger antioxidant or other beneficial effects on cerebral ischemia than caffeic acid are present in the higher MW fraction. Previous reports on the antioxidant effect of *P. linteus* indicated no strong antioxidant effect in the high MW fractions [7, 21, 22]. Taking these results together, the present study indicates the possibility that cerebral infarct reduction depends on mechanisms other than the antioxidative effect. In summary, we hypothesize that the antioxidant effect of caffeic acid in the low MW fraction together with other yet unidentified biologically active substances in the high MW fraction are responsible for the neuroprotective effect of *P. linteus* culture filtrate in cerebral ischemia.

Previous reports on the antioxidant effect of *P. linteus* were *in vitro* studies. On the other hand, the present study was an *in vivo* study using MCA occlusion rat models. It is possible that the high MW ingredients of *P. linteus* exert certain effects on the immune system *in vivo* and manifest their potential antioxidant effect. While the antioxidant activity of *P. linteus* has been reported to be due to caffeic acid which is contained in the lower MW fraction, the anti-inflammatory, anti-allergic and anti-tumor activities of *P. linteus* have been reported to be due to proteoglycans which is included in the higher MW fraction [7, 23, 24]. Nakamura et al. [7] reported that the main ingredient responsible for the strongest anti-tumor activity was proteoglycan, and they isolated *α*-1,3-glucan from the component with the strongest antitumor activity. Kim et al. [24] reported that a polysaccharide protein isolated from *P. linteus* stimulates tumoricidal cells such as macrophages and NK cells and induced the proliferation of B cells, which is the main mechanism of the antitumor activity of *P. linteus*. Moreover, Song et al. [25] reported that the antiangiogenic and antioxidant effects of *P. linteus* are partially responsible for its antitumor effect. The reduction of infarct volume induced by *P. linteus* culture extract is in part due to some ingredients present in the higher MW fraction. These ingredients may stimulate the immune system *in vivo*, and induce antioxidant effect to scavenge free radicals in the body. Furthermore, the present results also suggested that glycoprotein may play important and major roles in cortical infarct volume reduction induced by *P. linteus*. Further experiments are required to clarify these mechanisms.

## 5. Conclusions

In the present study, treatment with *P. linteus* culture filtrate resulted in significant reduction of cortical cerebral infarction in a rat permanent focal ischemia model and the effect was observed even by post-ischemic treatment. From the clinical point of view, these results suggest that *P. linteus* may be a promising new treatment approach for cerebral infarction with wide therapeutic time windows. Our finding that the higher MW fraction of the *P. linteus* culture filtrate demonstrated more prominent effect may provide a clue to identify the neuroprotective substances and mechanisms.
